# Exploring how individuals complete the choice tasks in a discrete choice experiment: an interview study

**DOI:** 10.1186/s12874-016-0140-4

**Published:** 2016-04-21

**Authors:** Jorien Veldwijk, Domino Determann, Mattijs S. Lambooij, Janine A. van Til, Ida J. Korfage, Esther W. de Bekker-Grob, G. Ardine de Wit

**Affiliations:** National Institute for Public Health and the Environment, Centre for Nutrition, Prevention and Health Services Research, PO Box 1 (internal postal code 101), 3720 BA Bilthoven, The Netherlands; Julius Center for Health Sciences and Primary Care, University Medical Center Utrecht, Utrecht, The Netherlands; Centre for Research Ethics and Bioethics, Uppsala University, PO box 564, SE-751 22 Uppsala, Sweden; Department of Public Health, Erasmus MC, University Medical Center Rotterdam, Rotterdam, The Netherlands; Health Technology and Services Research, University of Twente, Enschede, The Netherlands

**Keywords:** Discrete choice experiment, Methodology, Decision-making, Preference measurement, Testing assumptions, Interview, Think aloud

## Abstract

**Background:**

To be able to make valid inferences on stated preference data from a Discrete Choice Experiment (DCE) it is essential that researchers know if participants were actively involved, understood and interpreted the provided information correctly and whether they used complex decision strategies to make their choices and thereby acted in accordance with the continuity axiom.

**Methods:**

During structured interviews, we explored how 70 participants evaluated and completed four discrete choice tasks aloud. Hereafter, additional questions were asked to further explore if participants understood the information that was provided to them and whether they used complex decision strategies (continuity axiom) when making their choices. Two existing DCE questionnaires on rotavirus vaccination and prostate cancer-screening served as case studies.

**Results:**

A large proportion of the participants was not able to repeat the exact definition of the risk attributes as explained to them in the introduction of the questionnaire. The majority of the participants preferred more optimal over less optimal risk attribute levels. Most participants (66 %) mentioned three or more attributes when motivating their decisions, thereby acting in accordance with the continuity axiom. However, 16 out of 70 participants continuously mentioned less than three attributes when motivating their decision. Lower educated and less literate participants tended to mention less than three attributes when motivating their decision and used trading off between attributes less often as a decision-making strategy.

**Conclusion:**

The majority of the participants seemed to have understood the provided information about the choice tasks, the attributes, and the levels. They used complex decision strategies (continuity axiom) and are therefore capable to adequately complete a DCE. However, based on the participants’ age, educational level and health literacy additional, actions should be undertaken to ensure that participants understand the choice tasks and complete the DCE as presumed.

**Electronic supplementary material:**

The online version of this article (doi:10.1186/s12874-016-0140-4) contains supplementary material, which is available to authorized users.

## Background

A Discrete Choice Experiment (DCE) is a stated preference method in which individuals are asked to choose between two or more scenarios. Each scenario consists of several attributes with systematically varying levels that describe the product or service at hand. By monitoring individuals’ choices over a series of choice tasks, their preferences are elicited. DCEs are increasingly being used to make inferences on individuals’ preferences for a wide range of products or services within a health care context [1, 2].

DCE results are analyzed according to economic theory like Lancaster’s theory of demand [3], random utility theory [4, 5] or random regret minimization [6, 7]. These methodologies use a multi-attribute approach [8, 9]. It might be that individuals do not understand all the information that was provided to them and do not weigh all attributes when making their choices, especially not if risk information is included [10–12]. Therefore, this methodological approach may result in invalid conclusions regarding the attribute level estimates and the estimated potential uptake rates of goods or services. This in turn may lead to sub-optimal concordance between stated and revealed preferences. For these reasons, it is essential that researchers know how participants interpret the attributes and the levels, and ultimately make their decision.

### Theoretical assumptions

Conducting, analyzing and interpreting DCEs is based on several implicit and explicit assumptions regarding respondents’ decision-making, among which the ones listed next [13–15]. It is assumed that respondents are actively involved in completing the choice tasks. Additionally, respondents are expected to understand and interpret the information that they are provided with, as intended by the researcher [16, 17]. Finally, respondents are assumed to use complex decision strategies by considering all attributes and making their choice based on trade-offs between all attributes (continuity axiom) [7, 18, 19].

### Theoretical assumptions in practice

Both within and outside the health care setting, mainly quantitative research showed that these assumptions do not always hold. First, health-related DCEs often contain risk attributes. Research showed that respondents often misinterpret risk information [20, 21]. For example, respondents often interpret numerical values (ratio scales) as categorical information (for instance respondents recode a risk of 10 %, 30 % or 50 % to a low-medium-high risk) in DCEs [22–24]. In addition, respondents might apply simplified decision strategies such as choosing a scenario based on one attribute only [25]. Such simplifying strategies may especially be used by lower educated and less health literate respondents [8]. Second, completing choice tasks can be a cognitive challenge [26, 27]. Cognitively demanding decisions induce the use of simplified heuristics [28–31], which is not in accordance with the assumption that people use complex decision strategies; hence, people do not act in accordance with the continuity axiom. Additional research on this latter axiom showed that participants with dominant preferences base their decisions on one high priority attribute [32]. Such non-compensatory decision-making could either reflect a true strong preference for one specific attribute or it may be a way to avoid complex decision-making [33, 34]. Moreover, different quantitative studies show that up to 45 % of the participants have dominant preferences [33, 35, 36] and that lower educated participants more often base their decisions on dominant preferences [33]. Other studies showed that participants may disregard certain attributes and base their decision on some, but not on all attributes (attribute-non-attendance) [24, 32, 34, 37–41], thereby violating the continuity axiom.

### Aims

This study explored in depth how respondents complete choice tasks in a DCE, whether participants were actively involved, understood and interpreted the provided information correctly and whether they used complex decision strategies to make their choices and thereby acted in accordance with the continuity axiom. It was tested whether results differed by respondents’ educational level and health literacy. In contrast to other published qualitative studies that used a retrospective ‘top-down’ approach in relatively small samples to determine if and why respondents violate theoretical axioms, this paper uses a prospective ‘bottom-up’ approach in a large sample, and specifically focusses on respondents’ understanding and interpretation of risk information, and their use of complex decision-making strategies.

## Methods

### Discrete choice experiments

Two previously administered Dutch DCE questionnaires, that used a state-of-the-art approach by designing their experiment according to the latest guidelines for DCEs [13, 15], were used as case studies for the current study [42, 43]. One DCE reported on parental preferences for rotavirus vaccination while the other DCE reported on men’s preferences for prostate cancer-screening. Both DCEs selected their attributes and designed the survey based on formal literature review, interviews with experts, focus group discussions with participants, a pilot study and a think-aloud pilot study. Additionally, both DCEs contained several risk attributes, namely: vaccine effectiveness & frequency of severe side effects (rotavirus DCE) and proportion of unnecessary biopsies & proportion of unnecessary treatment (prostate cancer-screening DCE). Detailed descriptions of both studies are reported elsewhere [42, 43]. Since DCEs often cover very specific health topics, and thereby have very selective study samples, we included two DCEs to increase participant heterogeneity regarding demographic characteristics. A sample of the respondents of the case studies was re-contacted after previously indicating that they were willing to participate in further research. Participants completed the initial DCE at least 6 months before the interview. See Additional file 1 for a description of both studies, Tables 1 and 2 for a description of the included attributes and levels, and Additional file 2 for examples of choice tasks of both case studies.

**Table 1:** Attributes and levels for rotavirus DCE

Attributes	Explanation	Levels
Vaccine effectiveness	The percentage of children that will be protected against a rotavirus infection when vaccinated.	• 55 %
		• 75 %
		• 95 %
Frequency of severe side effects	The number of vaccinated children that will suffer from intussusception due to vaccination. Intussusception is an acute condition in which part of the bowel telescopes into another adjacent part of the bowel, resulting in obstruction [47]	• 1 in 10,000
		• 1 in 100,000
		• 1 in 1,000,000
Protection duration	The number of years that the vaccine protects against a rotavirus infection	• 1 year
		• 3 years
		• 6 years
Healthcare facility of vaccine administration		• Child welfare center
		• General practitioner
Out-of-pocket costs		• €0
		• €30
		• €140

**Table 2:** Attributes and levels for prostate cancer-screening DCE

Attributes	Explanation	Levels
Number of deaths from prostate cancer	It was given that 35 out of 1000 men die because of prostate cancer when no screening program is provided.	• 32 deaths (3 deaths prevented)
		• 28 deaths (7 deaths prevented)
		• 25 deaths (10 deaths prevented)
		• 18 deaths (17 deaths prevented)
Frequency of blood test		• Every year
		• Every 2 years
		• Every 3 years
		• Every 4 years
Number of unnecessary biopsies	Number of men, per 1000 men with an elevated PSA level, in which biopsies are unnecessary. Unnecessary biopsies were defined as biopsies in which no cancer was found, but in which PSA levels suggested that there was cancer.	• 200 unnecessary biopsies (800 justified biopsies)
		• 400 unnecessary biopsies (600 justified biopsies)
		• 600 unnecessary biopsies (400 justified biopsies)
		• 800 unnecessary biopsies (200 justified biopsies)
Number of unnecessary treatments	Number of men, per 1000 treated men, in whom treatment is unnecessary. Unnecessary treatment was defined as treatment that was not life prolonging, however it could lead to urine-loss and erection disorders due to treatment.	• 0 unnecessary treatments (1000 justified treatments)
		• 200 unnecessary treatments (800 justified treatments)
		• 500 unnecessary treatments (500 justified treatments)
		• 800 unnecessary treatments (200 justified treatments)
Out-of-pocket costs per year		• €0
		• €50
		• €100
		• €300

### Participants

In total, we included 70 participants for the current study; 35 from the rotavirus DCE and 35 from the prostate cancer-screening DCE. To study potential differences in decision-making strategies between lower and higher educated respondents, we purposively sampled equal proportions of lower and higher educated individuals from the participants of the previously performed DCE’s who had indicated to be willing to participate in future research. If subjects agreed to participate in the current study, they received a package with materials by mail. The Dutch National Ethics Board (Central Committee on Research involving Human Subjects) concluded that formal testing by a medical ethical committee was not necessary as participants only completed one non-invasive questionnaire on voluntary basis. Results were not analyzed or reported at the individual level, which is in accordance with the guidelines laid down in the Declaration of Helsinki.

### Interviews

Both face-to-face (*N* = 5 per cohort) and telephone interviews (*N* = 30 per cohort) were scheduled. Interview guides were developed for both DCEs. During a consensus meeting with all authors the categorization of answers was discussed. Although the topic of the two DCEs differed, both guides described a similar interview protocol to make the results of both groups comparable. The structured interviews were pilot tested (*N* = 7) to optimize the interview guide, to test the duration of an interview and to ensure both interviewers conducted the interviews in the same manner. This resulted in minimal adaptations to the interview guides. The final interview outline is described in Table 3. All interviews started with a short introduction to the current study. Next, participants were given some time to read the introduction of the DCE questionnaire. To get familiar with the DCE and the think aloud method, participants were asked to complete one choice task as a warm up exercise. The core of the interview consisted of three parts. During part one (think aloud part), participants completed four choice tasks from the original DCE (Table 3). We instructed the participants to think aloud when reading and completing the choice tasks. Part one of the interview took place without any specific guidance by the interviewers in order to mimic non-lab questionnaire completion situations as much as possible. However, if a participant was quiet for some time, the interviewer reminded him/her to keep thinking aloud and to report his/her thoughts. During part two of the interview (interview part), specific questions were asked to test the interpretation of the risk attributes, the understanding of the risk attributes, the decision strategy and the continuity axiom (Table 3). Finally, in part three of the interview, health literacy was measured both by means of a subjective self-reported questionnaire [44] and a validated objective measurement [44] (see Additional file 3). Results will be reported in the following order: choice task reading, interpretation of the risk attributes, understanding of the risk attributes, decision strategy and continuity axiom and differences by educational level and health literacy (stratified by the rotavirus and the prostate cancer-screening cohort).

**Table 3:** Interview outline

Short introduction to the current study including a choice task as a warm up exercise to get used to the DCE and thinking aloud		
Part 1: Think aloud part (categorization of participants observed decision-making behavior over four choice tasks, no specific questions asked)		
		Categorization options
Choice task reading	In which manner participants read the choice tasks	Attribute-wise
		Scenario-wise
		Directly motivating decision
		Otherwise
	Whether participants from the prostate cancer-screening cohort read the opt-out option aloud	Yes
		No
Interpretation of the risk attributes	How participants mentioned the risk attributes	Mentioning actual values
		Translating levels into ordinal scale
		Mentioning and interpreting values
Testing of continuity axiom	The number of attributes participants mentioned when motivating their decision for a certain scenario. Participants were marked as acting in accordance with the continuity axiom if they mentioned three or more attributes (i.e. less than the majority of the five included attributes) when motivating their decision	One
		Two
		Three or more
Decision strategy	The decision strategies participants applied to make their decision	Traded off attribute levels
		Based decision on one attribute
		Otherwise
Part 2: Interview part (asking direct questions)		
	Questions asked	Answer categories
Interpretation of the risk attributes	[^a^] was one of the characteristics that was included in the choice tasks. What did you have in mind with respect to this characteristic when you completed the choice tasks?	Exact definition
		Other definition
Understanding of the risk attributes	Please look at choice task x. If you were asked to make a choice based on [^a^] only, which scenario would you choose? This question was asked twice for all tested risk attributes (see^a^).	Scenario 1
		Scenario 2
		Don’t know
	Control question: Participants were asked to make a simple calculation with respect to the risk attributes to test their understanding of the numerical values of the risk attributes. For the rotavirus cohort: ‘Imagine, 1.000 children will get vaccinated with a vaccine that is 95 % effective. Assume that all children will get in contact with the virus. How many children will not get sick?’, and ‘Imagine, 300.000 children will get the rotavirus vaccine. Assume that the vaccine will lead to severe side effects in 1 out of every 100.000 children. How many children will suffer from severe side effects? For the prostate cancer-screening cohort: ‘Imagine a screening program in which out of 1.000 treatments, 200 are unnecessary. Imagine that 2.000 men participate in this screening program. How many men will be treated unnecessarily?’	Right answer
		Wrong answer
		Don’t know
Testing of continuity axiom	Those participants that based their decision on less than three attributes in all choice tasks were asked: ‘You included only x out of five characteristics when making your choice. Why was this the case?	Only one or two attributes important
		Hard to trade off multiple attributes
		Lack of attribute understanding
Part 3: Measuring health literacy^b^		
Subjective health literacy	Set of Brief Screening Questions (SBSQ-D) of Chew for prostate cancer-screening cohort only.	
	This instrument was already included in the initial rotavirus DCE and was therefore not repeated in the current study.	
Objective health literacy	Newest Vital Sign (NVS-D)	

^a^For the rotavirus cohort, these questions were asked for both the attributes vaccine effectiveness and frequency of severe side effects, while for the prostate cancer-screening cohort, these questions were only asked for the unnecessary treatment attribute since the levels of the two selected risk attributes (unnecessary treatment and unnecessary biopsy) were considered to be equal. ^b^See Additional file 3 for more information on these instruments

Two researchers (JV and DD) conducted the interviews. The interviewers used a predefined form to categorize reading and decision-making behavior in part one (this for instance entailed monitoring and marking how individuals read the choice tasks), as well as the answers the participants provided in part two and three of the interview (see Table 3). They also made notes and wrote down specific observations during each interview. Interviews were audio taped. Whenever there was doubt about participants’ behavior (in part one) or their answers (in part two), the two interviewers discussed and jointly listened to the audiotaped interview and completed the predefined form. As a result of this use of objective and pre-specified categories in the interviews, data could be analyzed with SPSS.

## Results

Table 4 describes the demographic characteristics of the participants who were interviewed. The average duration of the interviews in the rotavirus cohort was 27 min, while the average duration of the interviews in the prostate cancer-screening cohort was 41 min.

**Table 4:** Demographics of participants in both cohorts

		Rotavirus cohort (*n* = 35)	Prostate cancer-screening cohort (*n* = 35)
		Mean (SD)	Mean (SD)
Age in years		30.4 (4.5)	67.6 (5.5)
		Proportion (%)	Proportion (%)
Gender	Female	94.3	0
Education^a^	Lower	45.7	48.6
	Higher	54.3	51.4
Health literacy^b^	High subjective score	100	100
	High objective score	100	55.9

^a^Educational level was dichotomized into a higher and a lower educational level, whereby a Bachelor’s and/or Master’s degree were defined as a higher educational level and all other educational levels were defined as a lower educational level

^b^High subjective score includes participants with a score >2 on the SBSQ-D. High objective score includes participants with a score of 4–6 on the NVS-D

### Choice task reading

#### Think aloud part

Within both cohorts, the majority of the participants (60.7 % for the rotavirus cohort, and 56.4 % for the prostate cancer-screening cohort) read the choice tasks attribute-wise, starting from the top and moving to the bottom. In the rotavirus cohort, two other frequently used strategies for reading the choice tasks were 1) reading scenario-wise (15.0 %), and 2) directly motivating which of the two scenarios was preferred based on the attribute levels (14.3 %). This latter strategy was also often applied in the prostate cancer-screening cohort (18.6 %). Additionally, a considerable number of participants used different reading strategies (12.1 %); only reading attributes that were of personal importance, only reading attributes that differed between the two scenarios, and reading choice tasks (completely) in a random manner. The prostate cancer-screening choice tasks included an opt-out option (i.e. no screening), that was specifically read aloud by 42.9 % of the participants in choice task one, by 25.7 % in choice task two, 20.0 % in choice task three and 8.6 % of the participants in choice task four.

### Interpretation of the risk attributes

#### Think aloud part

With respect to the risk attributes, on average over all four choice tasks, 56.6 % of the participants of the rotavirus cohort mentioned the actual values of the attribute levels for vaccine effectiveness while completing the choice task and 45.9 % mentioned this for frequency of severe side effects. For the attribute ‘vaccine effectiveness’, on average over the four choice tasks, 17.5 % of the participants described the levels on an ordinal scale and 20.6 % combined reading with interpretation, like:

‘*In total 75 out of every 100 children are protected against a rotavirus infection, or three-quarters of the children do not become ill’*

With respect to the frequency of severe side effects, these percentages were 23.7 and 20.6 % respectively.

In the prostate cancer-screening cohort, 52.2 % of the participants mentioned the actual values of both of these attributes when reading the choice tasks. Additionally, 12.9 % of the participants interpreted the number of unnecessary biopsies and 14.3 % of the participants interpreted the number of unnecessary treatments when reading the choice tasks, for example:

*‘If I have to choose between 200 or 800 unnecessary biopsies/treatments, the likelihood of me having an unnecessary biopsies/treatment is four times as high in scenario two’*

Others did not mention these attributes while reading the choice tasks (30.7 % for the number of unnecessary biopsies and 29.3 % for the number of unnecessary treatments). Many of the participants experienced difficulties interpreting these two attributes. Some participants who experienced such difficulties did not understand the difference between biopsies and treatment, and some even thought they were similar or at least had similar side effects. For instance, participants stated:

*‘An unnecessary biopsy is an unnecessary treatment.’*

or

*‘Biopsy causes urine incontinence.’*

Some participants stated that they ignored these attributes when reading the choice tasks for those reasons, while others misinterpreted the numbers.

#### Interview part

Twenty percent of the participants of the rotavirus cohort was able to repeat the definition of vaccine effectiveness as described in the introduction section of the questionnaire. Another 57.1 % described vaccine effectiveness as ‘how well a vaccine works’ and 22.9 % provided a completely different definition. When asked about the meaning of the attribute side effects, the definition of side effects as provided in the questionnaire was mentioned by 37.1 % of the participants, 54.1 % interpreted side effects correctly but mentioned additional side effects that were not mentioned in the explanation of the attribute, such as a high temperature, feeling sick or dying, while 11.4 % provided a completely different definition.

In the prostate cancer-screening cohort, only 17.1 % of the participants was able to give the definition of the unnecessary treatment attribute as described in the attribute explanation section of the questionnaire.

### Understanding of the risk attributes

#### Interview part

All participants of the rotavirus cohort chose the vaccine with the highest effectiveness within both choice tasks when they were asked to choose based on this one attribute. On average over two choice tasks, all but three (4.3 %) participants chose the scenario with the lowest frequency of severe side effects. 77.1 % of the participants gave the correct answer to the control question for vaccine effectiveness, and 94.3 % of the participants gave the right answer to the control question for frequency of severe side effects. These results indicate that most participants were able to interpret percentages and frequencies correctly.

Within the prostate cancer-screening cohort, 83 % chose the screening option with the lowest level of unnecessary treatments. Although the concepts might not have been completely clear to some participants, 88.6 % answered the control question correctly, indicating that the participants were able to interpret the numbers of unnecessary treatment correctly.

### Decision strategy and continuity axiom

#### Think aloud part

In both cohorts, most participants mentioned the majority of the included attributes while motivating their choice for a scenario, which is in accordance with the continuity axiom (Table 5). In both cohorts, the majority also traded off between the levels of those attributes when motivating their decision, which again is in accordance with the continuity axiom. Within the rotavirus cohort, 20.0 % mentioned two attributes and 7.2 % only mentioned one attribute when motivating their decisions. In the prostate cancer-screening cohort 16.4 % mentioned two attributes, 17.9 % only mentioned one attribute and 5.7 % did not mention any of the attributes but chose to opt-out.

**Table 5:** Continuity axiom and decision strategy

		Average over all four choice tasks (%)
Rotavirus cohort (*n* = 35)	Motivating decision (continuity axiom)^a^	
	Motivation based on one attribute	7.2
	Motivation based on two attributes	20.0
	Motivation based on three or more attributes	72.9
	Decision strategy for those who acted in accordance with the continuity axiom	
	Traded off attribute levels between each other	85.6
	One attribute was most decisive	11.5
	Otherwise	2.9
Prostate cancer-screening cohort (*n* = 35)	Motivating decision (continuity axiom)^a b^	
	Motivation based on one attribute	17.9
	Motivation based on two attributes	16.4
	Motivation based on three or more attributes	60.0
	Decision strategy for those who acted in accordance with the continuity axiom	
	Traded off attribute levels between each other	60.0
	One attribute was most decisive	26.4
	Otherwise	13.6

^a^Participants were marked as acting in accordance with the continuity axiom, only if they motivated their decision based on three or more attributes

^b^These numbers do not add up to 100 % because some men did not mention any of the attributes when motivating which scenario they preferred; they chose opt-out (5.7 %)

#### Interview part

A total number of 16 participants (one in the rotavirus cohort and 15 in the prostate cancer-screening cohort) continuously traded off less than three attributes when completing the choice tasks. Nine out of those 16 participants stated that they traded off so few attributes because only those attributes were important to them, the other seven mentioned that they did so because they found it hard to trade off more attributes at once or because they did not understand the meaning of certain attributes. This latter category of seven participants comprised of participants for whom it is questionable whether they grasped the questions and understood the hypothetical nature of the choice tasks at all. The finding that some participants might not have understood the DCE at all is reflected in the fact that they decided per attribute which scenario they preferred, without making one final decision for one scenario. They also mentioned things such as:

*‘What is the difference between this question and the previous one?’*

or

*‘Can I switch between scenarios within one question?’*

### Differences by educational level and health literacy

Overall, there is a trend showing that more educated and literate participants included three or more attributes when motivating their decision and that they traded off between attributes more often compared to participants with a lower educational level or lower health literacy score (Table 6). Additionally, higher educated and literate participants more often correctly explained the risk attributes and more often answered the risk attribute control question correctly (Table 6). Finally, lower educated and less literate participants who based their decision on two attributes or less, more often stated that they found it difficult to compare all attributes.

**Table 6:** Differences in educational level and health literacy^a^

	Rotavirus cohort		Prostate cancer-screening cohort	
	Educational level (*n* = 35)^b^		Educational level (*n* = 35)^b^	
	Lower (%)	Higher (%)	Lower (%)	Higher (%)
Including three or more attributes when motivating decisions	81.3	100.0	70.6	83.3
Trading off attribute levels as a strategy to make a decision	56.3	73.7	35.3	44.4
Right explanation of vaccine effectiveness	12.5	26.3	-	-
Right explanation of severe side effects	56.3	94.7	-	-
Right explanation of unnecessary treatments	-	-	11.8	22.2
Right answer to control question on vaccine effectiveness	18.8	52.6	-	-
Right answer to control question on severe side effects	87.5	100.0	-	-
Right answer to control question on unnecessary treatments	-	-	82.4	94.4
			Health literacy (*n* = 34)^c^	
			Low (%)	High (%)
Including three or more attributes when motivating decisions			80.0	73.7
Trading off attribute levels to make a decision			33.3	47.4
Right explanation of unnecessary treatments			6.7	21.1
Right answer to control question on unnecessary treatments			80.0	94.7
			Combined measure (*n* = 20)^d^	
			Low (%)	High (%)
Including three or more attributes when motivating decisions			77.8	81.8
Trading off attribute levels to make a decision			33.3	54.5
Right explanation of unnecessary treatments			0.0	18.2
Right answer to control question on unnecessary treatments			77.8	100.0
Perceived it as difficult to trade off >2 attributes			60.0	33.3

^a^Differences in health literacy could only be calculated for the prostate cancer-screening cohort, because 100 % of the participants in the rotavirus cohort had high objective health literacy scores. ^b^Educational level was dichotomized into a higher and a lower educational level, whereby a Bachelor’s and/or Master’s degree were defined as a higher educational level and all other educational levels were defined as a lower educational level. ^c^High subjective score includes participants with a score >2 on the SBSQ-D. High objective score includes participants with a score of 4–6 on the NVS-D. ^d^Individuals that scored low on both educational level and objective health literacy (*n* = 9) or scored high on both educational level and objective health literacy (*n* = 11)

## Discussion

The majority of the participants preferred more optimal over less optimal attribute levels and answered the control question(s) regarding their understanding of the numerical values of the risk attributes correctly. At the same time, a large proportion of the participants was not able to repeat the exact definition of the risk attributes as explained to them in the introduction of the questionnaire. While the majority of the participants based their decision on three or more attributes by trading them against each other, which implies complex decision strategies and is in accordance with the continuity axiom, about a third of the participants used simplifying strategies such as basing their decision on less than three attributes.

Acting in contrast with the continuity axiom does not seem to be a problem per se. In real life, individuals might also not include all product characteristics when making their decision. However, within a DCE analysis, this may result in invalid conclusions regarding the attribute level estimates and estimated potential uptake rates of goods or services, since a multi-attribute approach is undertaken to analyze the data [8, 9]. This in turn may lead to sub-optimal concordance between stated and revealed preferences. This is also reflected by previous studies that indicated different DCE outcomes and significant influences on marginal rates of substitution depending on attribute-non-attendance being taken into account in DCE analyses [32, 39–41]. Previous research described that this non-compensatory decision-making behavior might have different causes; participants might actually have dominant preferences, it might be that the attribute levels are too similar, or that the participants lack understanding of certain attribute levels [18]. This latter was shown in the current study. In the rotavirus cohort for instance, 54 % of the participants mentioned that they had other and sometimes far more serious side effects in mind when completing the choice tasks. This will probably cause an overestimation of the relative importance of the side effects attribute, which affects the WTP estimate. Additionally, in the prostate cancer-screening cohort, a majority of the participants indicated that they did not understand one or more attributes (mostly the risk attributes). Studies state that a lack of understanding of certain attribute (levels) might be due to a lower educational level, older age and a lower health literacy [8, 21, 23, 45, 46]. The current study indeed showed that the number of attributes included in decision-making, decision strategy, interpretation of the risk attributes and understanding of the risk attributes differed between participants with different educational levels and health literacy scores. This might also be reflected by the fact that the mean interview duration of the less literate and older prostate cancer-screening group was almost 15 min longer compared to the rotavirus cohort. Besides educational level and health literacy scores, the topic of the DCEs and the included attributes and attribute levels may have added to the differences that were found between the two cohorts.

This study was subject to some limitations. Firstly, the two DCEs that we used as case studies for this study were quite complex, because each included two risk attributes. It is commonly known that the interpretation of such attributes is perceived as more difficult by participants than for instance qualitative attributes [20]. Difficulties in interpreting attribute levels and making decisions might therefore be more pronounced in this study compared to DCEs that include no or less risk-related attributes. However, since most health-related decisions include risk information, the case studies used for this study may be representative for many DCEs within a healthcare context. Secondly, this study focused on participants’ understanding of the provided information on risk attributes, their use of complex decision strategies and the continuity axiom. Other assumptions underlying the DCE methodology, namely the rationality assumption (which does not describe the psychological assumption of rationality, but merely represents the completeness and transitivity axioms) and the monotonicity axiom, were not tested. Thirdly, although this study used the well-recognized think aloud method for the interviews, additional methods such as eye-tracking might provide even more insight into how and what participants read. Such research could focus on visual attention sequences and underlying decision processes, as well as reading strategies regarding for instance the opt-out option. The current study showed a decrease in the percentage of respondents reading the opt-out option, which might reflect that participants assume this option to be fixed (attribute levels are not changing). Additionally, eye-tracking research will also provide insight in the potential discrepancy between the way participants complete a DCE with or without thinking aloud. Future research could incorporate such methods when investigating participants’ behavior when completing a DCE questionnaire. Fourthly, although efforts were made to mimic non-lab choice situations, the fact that the interviewers were present during DCE completion might have influenced how participants completed the choice tasks. Participants therefore might have been more committed to completing the DCE. As a result, we might have overestimated the number of participants that acts in accordance with the tested assumptions. Fifthly, the sample size of 70 is relatively large for an interview study, at the same time, this sample size is too small to draw any conclusions based on statistical testing. However, the trends in the findings and the agreement of the current findings with the existing literature related to educational level and health literacy (non-DCE studies) provide face validity for the current study results. Confirmation of our findings is needed, e.g. from new DCEs including (preferably objective) health literacy measurements as well as axiom testing questions in their study.

The results of our study indicate that respondents have difficulties understanding all the information that is provided to them, they do not always use complex decision strategies to make their choices and therefore do not always act in accordance to the continuity axiom. This was most prominent in respondents with a lower educational level, higher age and lower health literacy status. We therefore recommend to conduct DCE questionnaires among older and/or less health literate populations in, for instance, mini-labs, where participants complete DCEs in the presence of a researcher. Researchers have the opportunity to explain how to complete a DCE, including the hypothetical nature of the questionnaire and to answer questions that arise during the completion of the questionnaire, e.g. concerning the attributes and attribute levels. This is important especially among older target populations as participants in the prostate cancer-screening cohort sometimes indicated that they had difficulties interpreting the questions (e.g., *‘In real life, I have a blood test to check my PSA levels every year, so I can only choose a scenario with that frequency of blood testing’*). This is in line with the findings of previous studies [37, 38]. Moreover, when conducting online research, the understanding of attribute levels among participants with a lower educational level and/or health literacy can be enlarged by providing the option to include an explanation of the attributes by audio or other technical solutions, e.g. pop-ups when clicking on attributes or levels. In addition, the option to listen to the explanation again while completing the choice tasks could be offered. Another recommendation is that a thorough pilot testing phase is necessary while developing a DCE, which includes think aloud testing to a priori identify possible problematic issues with the completion of the questionnaire. Finally, age, educational level and health literacy should be standard measures to include in every DCE questionnaire as well as in the analysis of DCE data. Until options to correct DCE responses for possible differences in demographic characteristics become common practice, researchers should at least describe these measures in their population and explain the possible effects on the results retrieved.

## Conclusion

In conclusion, the majority of the participants seemed to have understood the provided information about the choice tasks, the attributes, and the levels. They used complex decision strategies (continuity axiom) and are therefore capable to adequately complete a DCE. However, based on the participants’ age, educational level and health literacy additional actions should be undertaken to ensure that participants understands the choice tasks and complete the DCE as presumed.

### Availability of data and materials

All relevant data are within the paper. Data will be made available via DANS: Data Archiving and Networked Services. The Dutch online archiving system Data Archiving and Networked Services (DANS), offers access to thousands of datasets in the humanities, the social sciences and other disciplines. Data will be stored for the long term and are accessible upon request. For further questions concerning license agreements please see www.dans.knaw.nl.

## Additional files

Additional file 1: Description of both studies, word document (DOC 33 kb) Additional file 2: Figure S1. Example of choice task rotavirus DCE, word document. **Figure S2.** Example of choice task prostate cancer-screening DCE, word document (ZIP 172 kb) Additional file 3: Description of the health literacy measures, word document (DOC 39 kb)
