# Supplementary material for: Exploring how individuals complete the choice tasks in a discrete choice experiment: an interview study
Source: BMC Med Res Methodol. 2016 Apr 21;16:45. doi: 10.1186/s12874-016-0140-4 (PMC4839138; doi:10.1186/s12874-016-0140-4)
Supplement: Additional file 1: — Description of both studies, word document (DOC 33 kb) [file 12874_2016_140_MOESM1_ESM.doc]

**Additional file 1:** Description of both studies

*Rotavirus DCE*

Between January and March 2013, Veldwijk et al. conducted a DCE on parental preferences for rotavirus vaccination of newborns in the Netherlands. A random sample of 2500 parents with newborns was selected from the Praeventis database (a national vaccination register in which the vaccination status of all Dutch newborns is registered) to participate in this study. The DCE questionnaire consisted of nine choice tasks. In the choice tasks, participants were asked to choose between two different rotavirus vaccine scenarios to protect their child from an infection. Each choice task was constructed based on five attributes with either two or three levels (Table 1a). In total, 959 participants completed the questionnaire, of which 202 gave permission to be re-contacted for further research.

*Prostate cancer screening DCE*

To investigate men’s preferences and trade-offs for prostate cancer screening, de Bekker-Grob et al. conducted a DCE between January and May 2011 among a population-based random sample of 1000 men aged 55 to 75, living in the Rijnmond region of the Netherlands. The DCE questionnaire comprised 16 choice tasks, in which participants were asked to choose between a no screening scenario (opt-out) and two prostate cancer-screening scenarios. Each scenario consisted of five attributes, with each four levels (Table 1b). In total, 459 men responded to the questionnaire and 373 gave permission to be contacted again for additional questions.

**References**

1 Veldwijk J, Lambooij MS, Bruijning-Verhagen P, Smit HA, De Wit GA, Parental preferences for rotavirus vaccination in young children: a Discrete Choice Experiment. Vaccine. 2014;32:6277-83.

2 de Bekker-Grob EW, Rose JM, Donkers B, Essink-Bot ML, Bangma CH, Steyerberg EW, Men's preferences for prostate cancer screening: a discrete choice experiment. Br J Cancer. 2013;108:533-41.
