# Supplementary material for: Exploring how individuals complete the choice tasks in a discrete choice experiment: an interview study
Source: BMC Med Res Methodol. 2016 Apr 21;16:45. doi: 10.1186/s12874-016-0140-4 (PMC4839138; doi:10.1186/s12874-016-0140-4)

**Additional file 2, figure 1**: Example of choice task rotavirus DCE

Imagine that a vaccine against rotavirus infections would become available within the Netherlands. In what situation would you prefer to vaccinate your newborn, situation 1 or situation 2?


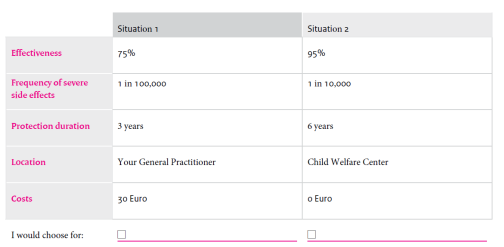

Supplement: Additional file 2: Figure S1. — Example of choice task rotavirus DCE, word document. Figure S2. Example of choice task prostate cancer-screening DCE, word document (ZIP 172 kb) [file 12874_2016_140_MOESM2_ESM.zip › Additional file 2/Additional file 2, figure 1R2.docx]
