# Supplementary material for: Exploring how individuals complete the choice tasks in a discrete choice experiment: an interview study
Source: BMC Med Res Methodol. 2016 Apr 21;16:45. doi: 10.1186/s12874-016-0140-4 (PMC4839138; doi:10.1186/s12874-016-0140-4)
Supplement: Additional file 2: Figure S1. — Example of choice task rotavirus DCE, word document. Figure S2. Example of choice task prostate cancer-screening DCE, word document (ZIP 172 kb) [file 12874_2016_140_MOESM2_ESM.zip › Additional file 2/Additional file 2, figure 2R2.docx]

**Additional file 2, figure 2**: Example of choice task prostate cancer-screening DCE

Thirty-five out of every 1000 deaths among men are caused by prostate cancer. Which alternative do you prefer to reduce your risk of dying from prostate cancer: no screening, screening program 1, or screening program 2? *(please, tick one box)*

|  | **No screening** | **Program 1** | **Program 2** |
| --- | --- | --- | --- |
| **Amount of men per 1.000** men who will **die** from prostate cancer | **35 deaths**  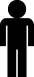  (0 deaths prevented) | **25 deaths**  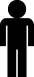  (10 deaths prevented) | **18 deaths**  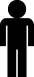  (17 deaths prevented) |
| **Frequency** of a blood **test** | **No** blood test | Every **4 years** a blood test | Every **3 years** a blood test |
| **Amount of men per 1,000** men with an increased PSA who receive an **unnecessary biopsy**  (= no cancer detected, although the blood test suggested that a blood test was necessary) | **Not applicable** | **400 unecessary biopsies**  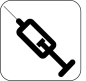  (600 correct biopsies) | **800 unnecessary biopsies**  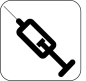  (200 correct biopsies) |
| **Amount of men per 1,000** treated men who receive an **unnecessary treatment**  (= no increase in life expectancy, but there is a risk of urine incontinence and erection problems due to treatment) | **Not applicable** | **0 unnecessary treatments**  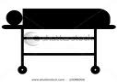  (1,000 correct treatments) | **500 unnecessary treatments**  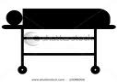  (500 correct treatments) |
| **Out of pocket cost** per year during the period of the screening program | **0 euro per year** | **100 euro per year**  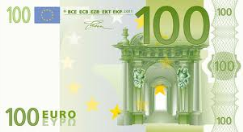 | **50 euro per year**  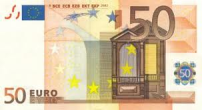 |
| **Which alternative** **would you choose?** |  |  |  |
