# Supplementary material for: Exploring how individuals complete the choice tasks in a discrete choice experiment: an interview study
Source: BMC Med Res Methodol. 2016 Apr 21;16:45. doi: 10.1186/s12874-016-0140-4 (PMC4839138; doi:10.1186/s12874-016-0140-4)
Supplement: Additional file 3: — Description of the health literacy measures, word document (DOC 39 kb) [file 12874_2016_140_MOESM3_ESM.doc]

**Additional file 3:** Description of the health literacy measures

The prostate cancer screening cohort was asked to complete the three validated Dutch questions of the Set of Brief Screening Questions (SBSQ-D) of Chew to measure their subjective health literacy. This instrument was already included in the initial rotavirus DCE, these questions were therefore not repeated in the current study. The SBSQ-D contains questions on how often participants need help to read letters from their GP/specialist, how sure participants are that they complete medical forms correctly and how often participants find it difficult to find information about their health. Participants scored these questions on a 5-point Likert scale, from zero to four. An average score of ≤2 indicates inadequate health literacy, while an average score >2 indicates adequate health literacy . The Dutch version of the Newest Vital Sign (NVS-D) was included as an objective measure of health literacy for both groups. To measure the participants’ health literacy status, they were asked six questions about an ice cream nutrition label. Participants scored one point for each correctly answered question, with a maximum of 6 points. A score of 4-6 indicates adequate health literacy .

**Reference**

1 Fransen MP, Van Schaik TM, Twickler TB, Essink-Bot ML, Applicability of internationally available health literacy measures in the Netherlands. Journal of health communication. 2011;16 Suppl 3:134-49.
